# Common Disconnections in Glioma Surgery: An Anatomic Description

**DOI:** 10.7759/cureus.1778

**Published:** 2017-10-16

**Authors:** Chad Glenn, Andrew K Conner, Meherzad Rahimi, Robert G Briggs, Cordell Baker, Michael Sughrue

**Affiliations:** 1 Department of Neurosurgery, University of Oklahoma Health Sciences Center

**Keywords:** diffusion tractography, glioma, awake mapping, lobectomy, white matter tracts, dti

## Abstract

Within the surgical treatment of glioma, extended survival is predicated upon extent of resection which is limited by proximity and/or invasion of eloquent structures. Diffusion tensor imaging (DTI) tractography is a very useful tool for guiding supramaximal surgical resection while preserving eloquence. Although gliomas can vary significantly in size, shape, and invasion of functionally significant brain tissue, typical surgical disconnection patterns emerge. In this study, our typical surgical paradigm is outlined. We describe our surgical philosophy for resecting gliomas supramaximally summarized as define, divide, and destroy with the adjuvant utilization of neuronavigation and DTI. We describe the most common disconnections involved in glioma surgery at our institution; specifically, delineating tumor disconnections involving the medial posterior frontal, lateral posterior frontal, posterior temporal, anterior occipital, medial parietal, and insular regions. Although gliomas are highly variable, common patterns emerge in relation to the necessary disconnections required to preserve eloquent brain while maximizing the extent of resection.

## Introduction and background

The intrinsic properties of gliomas necessitate careful preoperative planning. The treatment goal in any glioma surgery is to safely remove as much tumor as possible [1-12], and achievement of this goal is dependent upon the correct conceptualization of the margins between the tumor and eloquent functional networks [13-14]. Defining these boundaries is a cognitive task that incorporates anatomic knowledge and radiographic interpretation. 

Diffusion tensor imaging (DTI) tractography does not tell us where the functional networks are located per se, but tractography does suggest where large bundle white matter tracts are likely to be located [15]. As gliomas commonly expand gyri and later spread along white matter tracts [16], they may be classified not only by their anatomic location but also by which white matter tract they have overrun [16-17]. By determining the pattern of spread and, therefore, the tracts likely to be involved, it is possible to develop a neuroanatomic understanding of the tumor.

**Figure 1:**
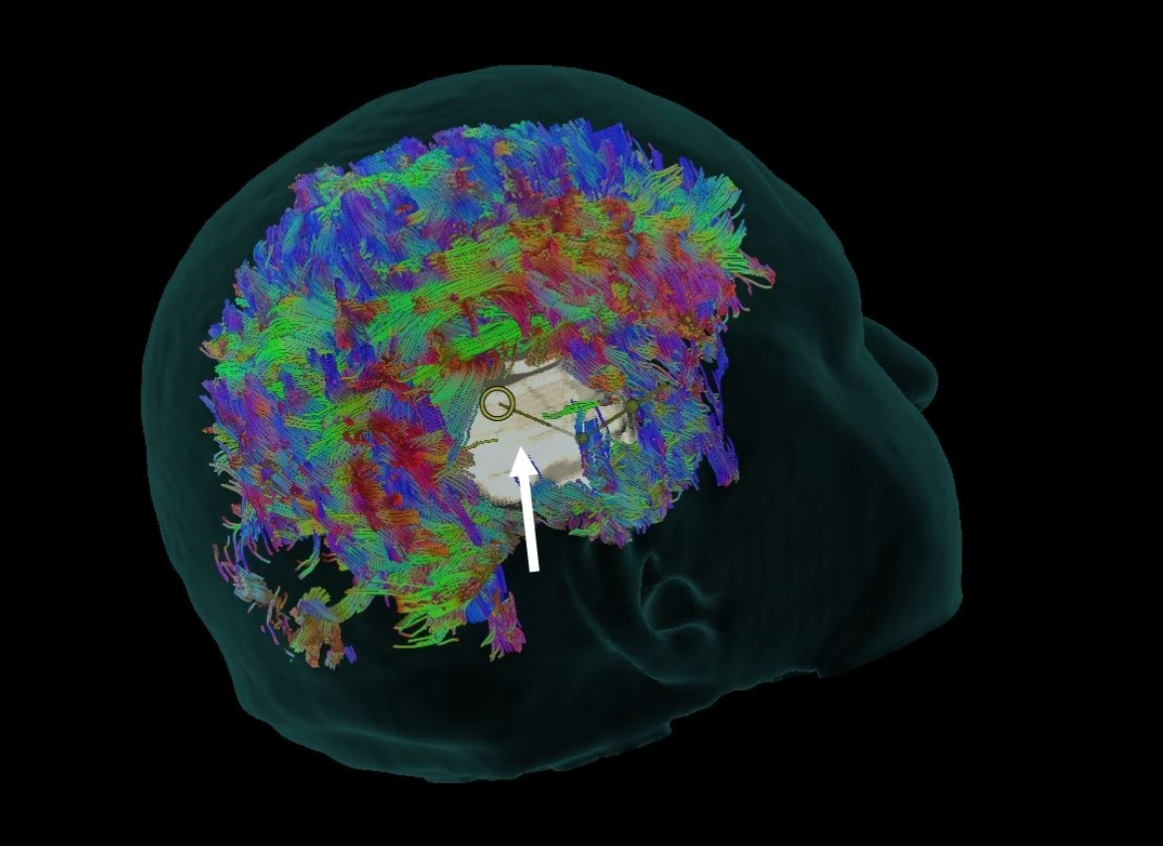
Whole Brain Tractography Synaptive BrightMatter™ glass head preoperative analysis using whole brain tractography to show neighboring and intersecting white matter tracts at the brain-tumor interface. The white arrow points to a computer-generated tumor model; note the surrounding white matter tractography.

Initially, the most difficult aspect in planning a glioma surgery is developing a three-dimensional conceptualization of the tumor and its neighboring structures. When coupled with an abundance of tractography data, the model can become quite complex [17-18]. With the use of whole brain tractography, a preoperative analysis of neighboring and/or intersecting white matter tracts at the brain-tumor interface may be performed (Figure 1). By identifying involved tracts preoperatively, one may tailor the approach in such a way as to avoid fiber transgression.

DTI, when used in conjunction with awake cortical and subcortical mapping, can be a very useful guide in localizing eloquent structures based on the commonly known functional significance of individual white matter tracts [17-18]. Furthermore, gliomas in certain locations are amenable to predictable methods of anatomic disconnection [4]. Here, we attempt to provide an anatomic overview of the common disconnections we perform at our institution.  

## Review

Glioma surgery defined

A simplified approach to glioma surgery can be summed up as follows: define, divide, and destroy. Here, we will illustrate our surgical method by explaining the necessary details to successfully achieve each of these principles.

Define

The first step in successfully removing a glioma to a functionally significant limit (supramaximal resection) is to define the eloquent boundaries associated with the mass. Although these areas are somewhat variable between patients and influenced by mass effect and functional reorganization [3], in our experience, they tend to follow general patterns based on white matter tractography. Defining the boundaries of a planned resection with the goal of a supramaximal resection is begun with the utilization of DTI to localize the likely areas of cortical eloquence, such as cortical motor function in relation to the cortical termination of the corticospinal tract (CST), speech/naming sites with the posterior temporal cortical termination of the superior longitudinal fasciculus (SLF), and expressive speech sites in conjunction with the frontal cortical termination of the inferior frontal-occipital fasciculus (IFOF), among others. We have found that these described cortical terminations are highly correlated with eloquence at the cortical surface [15]. Once this is complete, the second step to defining the operative corridor or planned resection is to define the subcortical portion of the glioma in conjunction with the surrounding white matter tracts, i.e., the SLF, IFOF, CST. These boundaries create the framework for the craniotomy leading to the expected resection cavity and are tailored to patients based initially by DTI imaging. After exposure of the brain, we utilize negative awake brain mapping, as described by others [19-20], to determine whether the planned disconnection is feasible within the functional constraints of the patient. This is first carried out on the cortical surface followed by subcortical identification of eloquent white matter tracts in conjunction with awake double-task mapping [21-22]. Positive sites are marked and protected sequentially.

Divide

After defining cortically eloquent sites, a surgical division is then carried out to the subcortical level. As stated in the definition phase, a subcortical division is undertaken while the patient is performing a double task, taking pause to evaluate for eloquence with direct electrical stimulation prior to performing disconnection with the aid of DTI enhanced neuronavigation. Thus, this portion is carried out nearly simultaneously with the definition phase. Typically, a subcortical disconnection is considered complete once the ventricle is encountered (i.e., frontal horn in frontal lobectomy or temporal horn in temporal lobectomy), or an intersecting white matter tract is encountered, prohibiting further division or disconnection (i.e., IFOF in frontal lobectomy).

Destroy

Once the boundaries of the glioma/eloquence interface are defined and the mass is divided or disconnected from eloquent brain tissue, the patient is sedated and the now surgically disconnected non-eloquent brain tissue is removed nearly en bloc. Typically, this step involves completion of disconnections to the structural boundaries of the fossa housing the affected area of the brain, for instance, the floor of the middle fossa in a temporal lobectomy. 

Common Neoplasm/Eloquence Disconnections

With the aforementioned steps that are taken to achieve supramaximal resection of gliomas, we utilize the Synaptive BrightMatter™ (Synaptive Medical, Inc., Toronto, Canada) planning software to illustrate the typical disconnections that are encountered in common glioma surgery.

As this is a retrospective anatomical analysis, informed consent was not required by the University of Oklahoma Health Sciences Center Institutional Review Board (IRB); use of this data was approved via IRB #3199.

Disconnections

Medial Posterior Frontal

**Figure 2:**
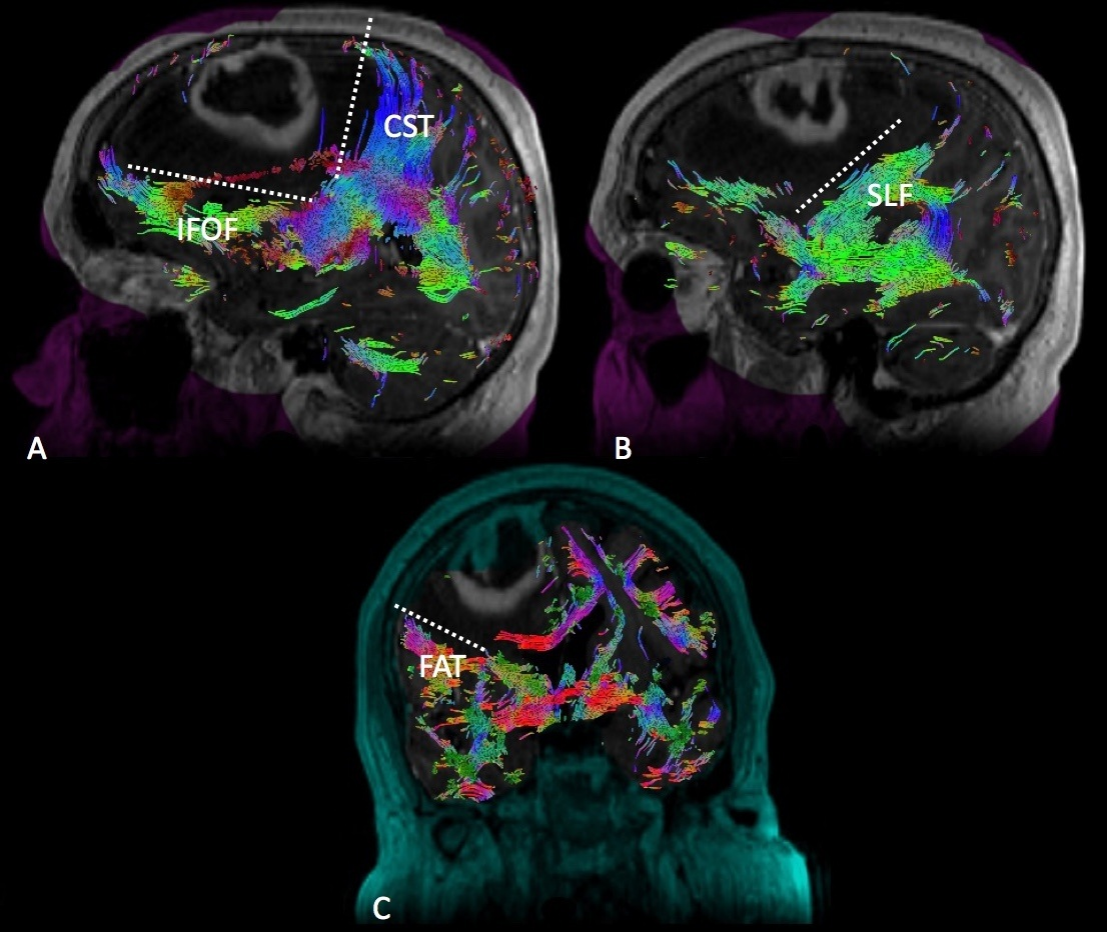
Medial Posterior Frontal Diffusion tractography enhanced T1 magnetic resonance imaging (MRI) with contrast with (A) sagittal, (B) sagittal, and (C) coronal views demonstrating a medial posterior frontal glioma in relation to the surrounding white matter tracts. Labeled white matter tracts associated are shown as follows: inferior fronto-occipital tract (IFOF), corticospinal tract (CST), superior longitudinal fasciculus (SLF), and frontal aslant tract (FAT). The disconnection pattern is shown as a white dotted line.

Tumors occupying the medial posterior frontal lobe must be disconnected from both the motor and speech networks (Figure 2). Resection of this tumor type tends to generate an L-shaped cut with the motor system located posteriorly with respect to the CST and frontal aslant tract (FAT) posterolaterally, as well as the speech networks laterally with respect to the SLF and IFOF. The medial aspect of the disconnection extends from the midline to the falx. At inferior depth, the resection continues into the frontal horn of the lateral ventricle, not to extend posteriorly beyond the head of the caudate, thus completing the inferior aspect of the disconnection.

Lateral Posterior Frontal

**Figure 3:**
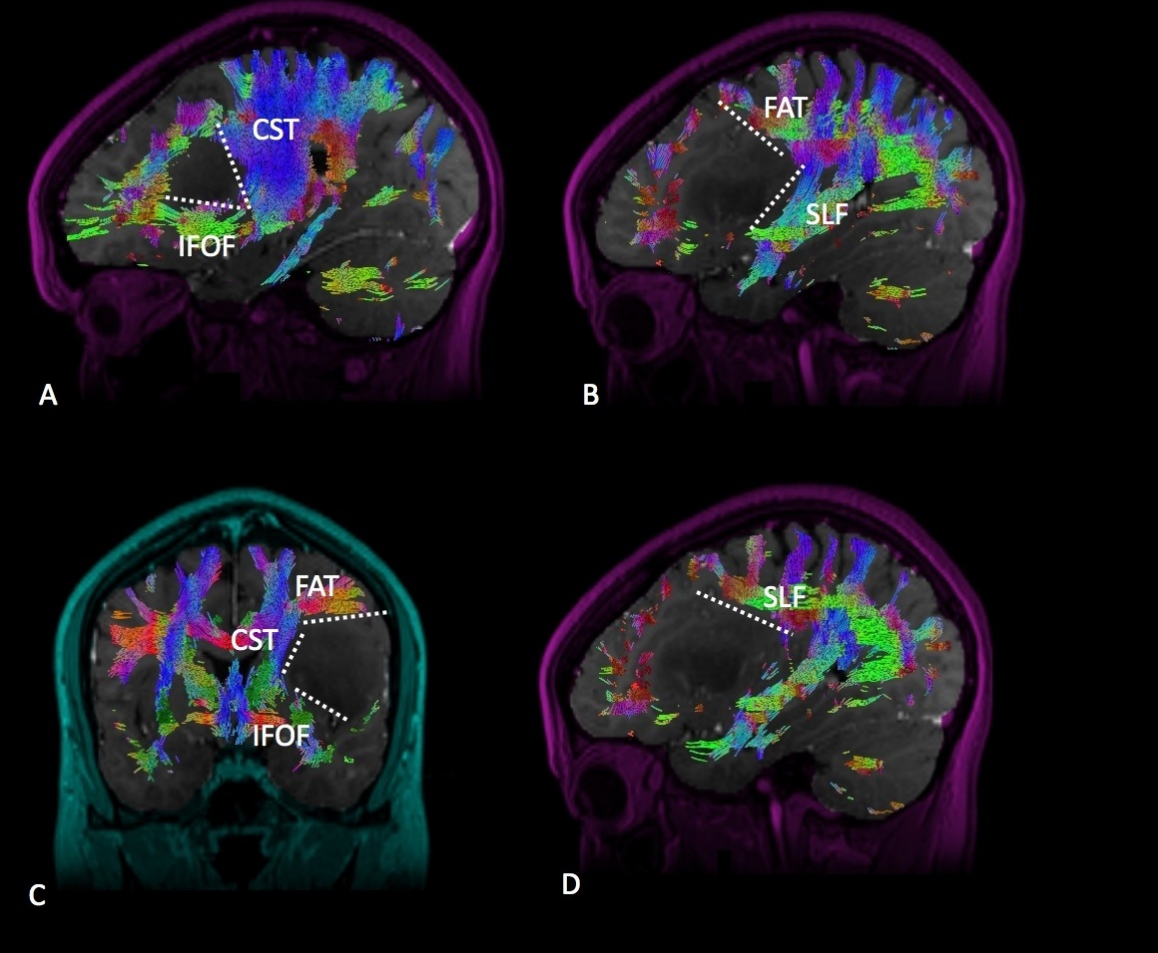
Lateral Posterior Frontal Diffusion tractography enhanced T1 magnetic resonance imaging (MRI) with contrast with (A) sagittal, (B) sagittal, (C) coronal, and (D) sagittal views demonstrating a lateral posterior frontal glioma in relation to the surrounding white matter tracts. Labeled white matter tracts associated are shown as follows: inferior fronto-occipital tract (IFOF), corticospinal tract (CST), superior longitudinal fasciculus (SLF), and frontal aslant tract (FAT). The disconnection pattern is shown as a white dotted line.

Tumors occupying the lateral posterior frontal lobe require disconnection from the speech networks located posteriorly, such as the SLF. Resection of this tumor type tends to generate a J-shaped cut with the concave aspect of the J facing laterally in a coronal plane (Figure 3). The superior aspect of the disconnection typically hugs the FAT. The medial disconnection is limited by the deep anatomy, either the insula or, deeper to that, the basal ganglia/CST complex. The inferolateral disconnection is limited by the IFOF as it exits the sub-insular space traveling to its frontal cortical termination. While there is not a reliable landmark at depth, the basal ganglia should not be transgressed. Following the convexity, the lateral and inferior margins of the cut extend to the Sylvian fissure or possibly the opercular pia. 

Posterior Temporal

**Figure 4:**
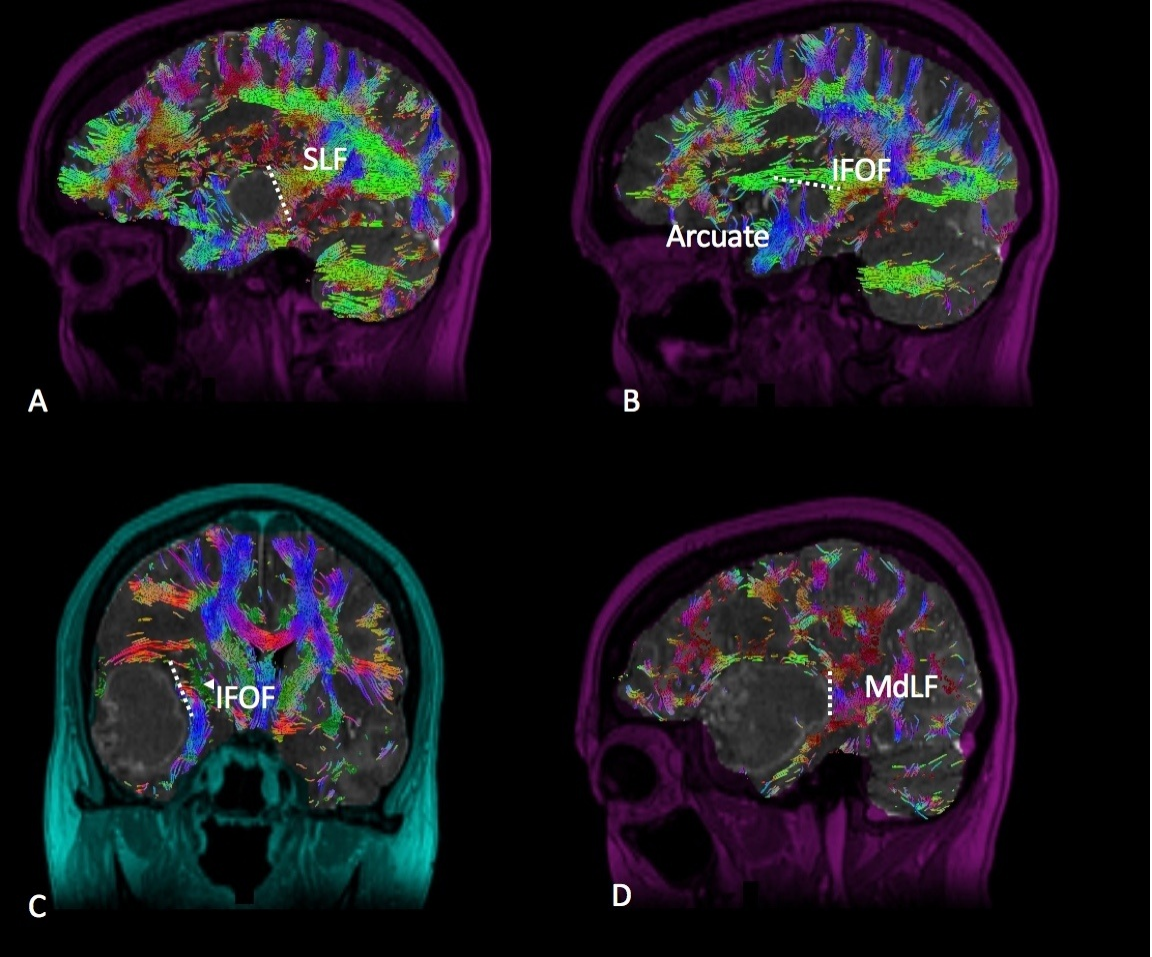
Posterior Temporal Diffusion tractography enhanced T1 magnetic resonance imaging (MRI) with contrast with (A) sagittal, (B) sagittal, (C) coronal, and (D) sagittal views demonstrating a posterior temporal glioma in relation to the surrounding white matter tracts. Labeled white matter tracts associated are shown as follows: inferior fronto-occipital tract (IFOF), superior longitudinal fasciculus (SLF), middle longitudinal fasciculus (MdLF), and arcuate fasciculus (Arcuate). The disconnection pattern is shown as a white dotted line.

Tumors occupying the posterior aspect of the temporal lobe must be disconnected from the SLF posteriorly and the IFOF at depth in the sub-insular region (Figure 4). The middle longitudinal fasciculus (MdLF) in the superior temporal gyrus, typically located just anterior to the SLF, should also be recognized and help guide the posterosuperior disconnection. Resection of this tumor type generates a straight vertical cut along the posterior boundary of the disconnection. The medial extent of this cut involves a sub-pial dissection of the superior temporal gyrus, thus protecting the vessels within the Sylvian fissure and the insula. At depth, the temporal horn of the lateral ventricle is opened, providing a useful anatomic landmark for access to the medial temporal lobe structures and defining completion of the disconnection.

Anterior Occipital

**Figure 5:**
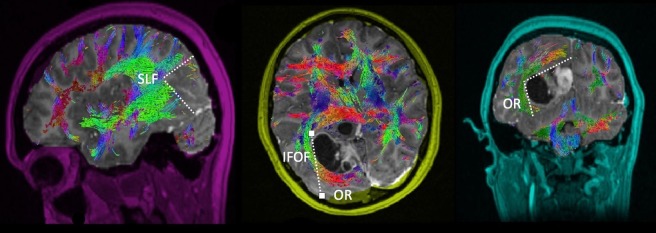
Anterior Occipital Diffusion tractography enhanced T1 magnetic resonance imaging (MRI) with contrast with (A) sagittal, (B) axial, and (C) coronal views demonstrating an anterior occipital glioma in relation to the surrounding white matter tracts. Labeled white matter tracts associated are shown as follows: inferior fronto-occipital tract (IFOF), superior longitudinal fasciculus (SLF), and optic radiations (OR). The disconnection pattern is shown as a white dotted line.

Tumors occupying the anterior aspect of the occipital lobe or occipitotemporal region must be disconnected from the SLF anterolaterally (Figure 5). Resection of this tumor type will generate an inverted L-shape if the optic radiations are to be spared or in some cases of compromised visual fields; the optic radiations may be transgressed to achieve supramaximal resection as seen in an occipital lobectomy. The medial aspect of this cut is extended midline to the falx. At depth, the atrium of the lateral ventricle provides a useful landmark as well as defining the completion of the planned disconnection. The IFOF can be avoided in most cases by staying in the medial portion of the atrium as it runs in the tempo-parietal-occipital junction lateral to the atrium.

Medial Parietal

**Figure 6:**
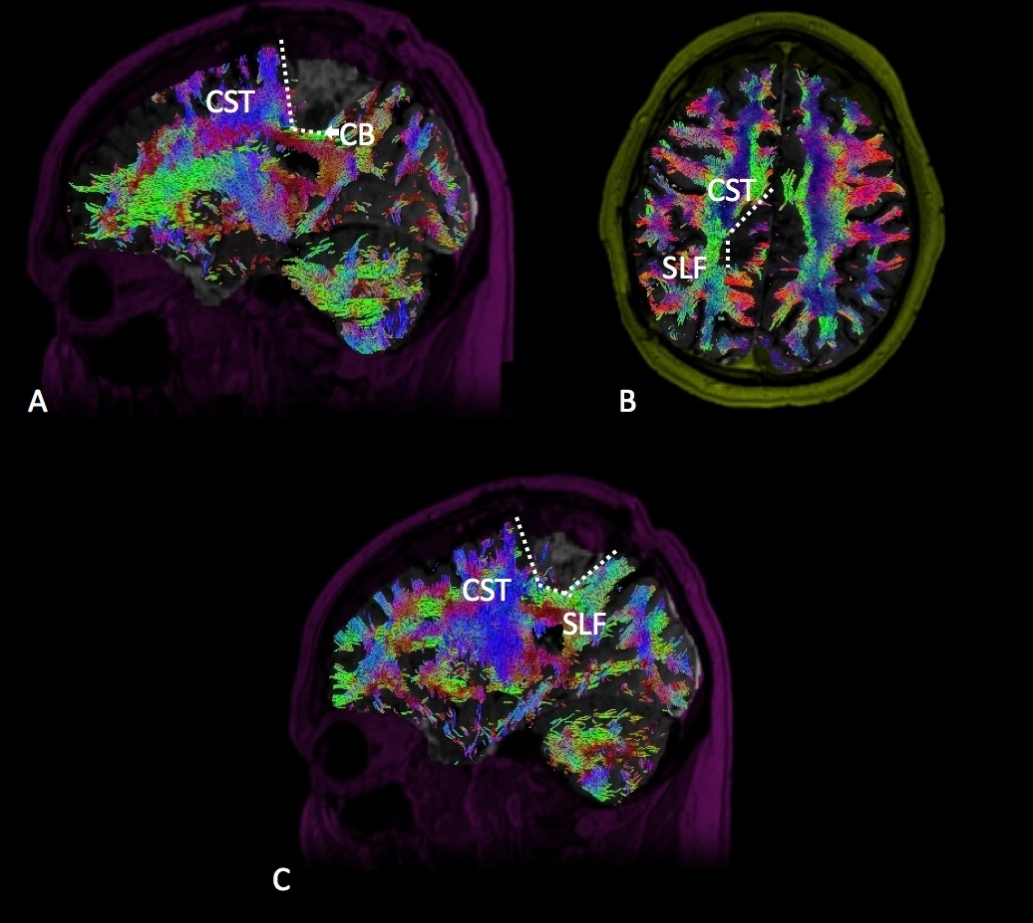
Medial Parietal Diffusion tractography enhanced T1 magnetic resonance imaging (MRI) with contrast with (A) sagittal, (B) axial, and (C) sagittal views demonstrating a medial parietal glioma in relation to the surrounding white matter tracts. Labeled white matter tracts associated are shown as follows: superior longitudinal fasciculus (SLF), corticospinal tract (CST), and cingulate bundle (CB). The disconnection pattern is shown as a white dotted line.

Tumors occupying the medial parietal lobe must be disconnected from the sensorimotor networks anteriorly, i.e., CST, and the SLF, and in some cases of more inferolateral involvement, portions of the IFOF laterally and inferiorly (Figure 6). The series of disconnections performed generates a square-shaped cut along the cortex with the anterior disconnection located posteriorly to the sensorimotor system and the posterior disconnection overlying the inferior parietal lobule. Both the anterior and posterior cuts are extended medially to the falx. The lateral disconnection overlies the SLF and possibly a portion of the IFOF, dependent on the depth of tumor invasion. At depth, the cuts converge at the atrium of the lateral ventricle.  

Insula

**Figure 7:**
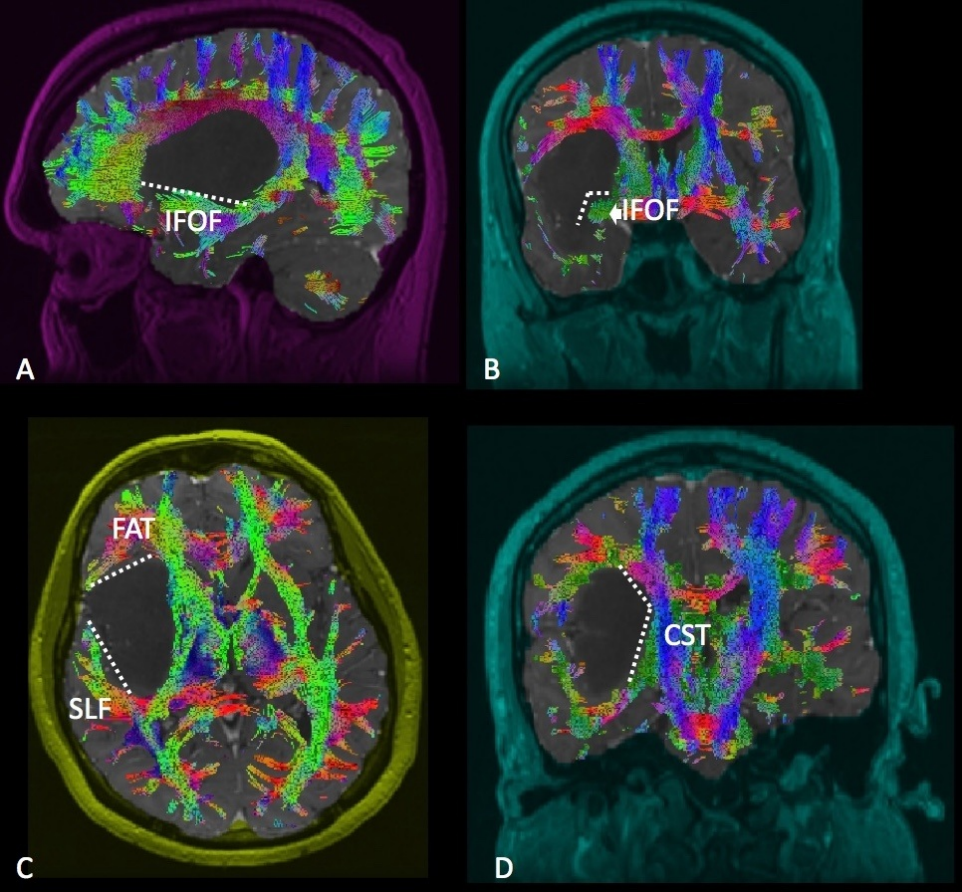
Insula Diffusion tractography enhanced T1 magnetic resonance imaging (MRI) with contrast with (A) sagittal, (B) coronal, (C) axial, and (D) coronal views demonstrating an insular glioma in relation to the surrounding white matter tracts. Labeled white matter tracts associated are shown as follows: superior longitudinal fasciculus (SLF), corticospinal tract (CST), inferior fronto-occipital tract (IFOF), and frontal aslant tract (FAT). The disconnection pattern is shown as a white dotted line.

Adequate visualization of the insula generally follows completion of a posterior temporal disconnection. We have found this to be necessary in order to properly visualize the insular boundaries. Resection of insular tumors requires disconnection from the IFOF, SLF, and motor fibers while avoiding entry into the basal ganglia and internal capsule (Figure 7). Anteriorly and inferiorly along the limen insulae, disconnection from the IFOF is performed. The SLF and CST provide boundaries for the superior disconnection. Antero-superiorly, the FAT is encountered and can guide this portion of the resection. Exposure of the hippocampus at the time of the posterior temporal disconnection provides a useful depth landmark, given that it is typically in the coronal plane with the basal ganglia. 

Discussion

A description of a glioma’s anatomic location within the gyri and/or along white matter tracts is an important initial step in understanding what critical structures are nearby [4, 16]. Completion of this cognitive task begins with the understanding of what disconnection is likely to be required and what tracts are likely to be encountered. Whole brain tractography allows the surgeon to construct a three-dimensional model of the tumor and the surrounding white matter tracts preoperatively [4, 17-18]. Once the tumor and its surrounding structures are defined, various approach trajectories may be trialed until the optimal working angle is identified [17]. Our aim with this anatomical study is to illustrate the commonly encountered cortical/subcortical disconnections relevant in supramaximal glioma resection.

Although we have described a basic overview of the common disconnection techniques that we perform, there are many variations as is the nature of glioma surgery. By visualizing where white matter tracts are likely invaded or displaced, these techniques may be tailored to patients and further refined with adjunct awake mapping techniques [17-18]. Obviously, there are many instances in which a glioma is not conveniently packaged. For example, gliomas of the temporoparietal junction, motor system, or those in the deep medial structures do not follow a simplified pattern of disconnection and instead require constant vigilance during operative planning, division, and removal [4, 18]. We tend to consider these tumors as “surrounded” by eloquent structures. In these situations, whole brain tractography may reveal small windows that offer the safest approach option [17]. It is not uncommon at our institution to expose a larger operative corridor to accommodate a few different options for trajectory into the mass in question. An example of this type of situation is relevant within motor strip gliomas to expose the anterior, posterior, and superior trajectory into the mass, given that an entry planned through an assumed silent cortical area may map positive and thus be avoided. This is the most apparent with lesions in and seemingly surrounded by function. 

The advantages of whole brain tractography on aiding in approach selection and intraoperative navigation are noteworthy. However, we tend to prefer utilizing a combination of tractography in conjunction with cortical and subcortical mapping techniques. In our experience, the identification of a white matter tract of presumed eloquence on tractography does not necessarily obviate resectability. Ultimately, resection is permitted by defining a quiet or non-eloquent area confirmed with direct cortical stimulation. However, by using tractography in synchrony with neuronavigation, we are able to repeatedly reference the proximity to involved/intersecting white matter tracts to guide planned maneuvers required for tumor resection. Both awake mapping and the utilization of DTI in conjunction lend themselves well to one another, as cortical and subcortical mapping can support or in some cases refute expected areas of eloquence. Overall, we find that DTI is more or less a roadmap that helps identify the major boundaries between the tumor and brain interface. Certainly, tractography is a very useful tool in planning an approach aimed at maximal resection and complements awake mapping and vice versa. We feel this technique to be a safety net to minimize the risk of inadvertent fiber transgression subcortically and a valuable tool in predicting eloquence upfront. 

## Conclusions

The recognition that gliomas generally spread along white matter tracts allows for their description. Common glioma disconnection techniques, along with a description of the involved white matter tracts, are discussed. We think there is great value in combining these techniques with whole brain tractography. This allows the surgeon to visualize the intersecting white matter tracts’ relationship to the tumor margin and tailor the disconnection technique to the individual tumor in order to maximize tumor resection as well as preserve eloquence.
